# Genome-Wide Characterization of the *HSF* Gene Family in *Paulownia fortunei* and Preliminary Functional Analysis of *PfHSF6* in Drought Response

**DOI:** 10.3390/ijms27209338

**Published:** 2026-10-21

**Authors:** Jie Liu, Chunyang Liu, Linhuan Wang, Zhenli Zhao, Yabing Cao

**Affiliations:** 1 Institute of Paulownia, Henan Agricultural University, Zhengzhou 450046, China; 2 College of Forestry, Henan Agricultural University, Zhengzhou 450046, China

**Keywords:** *Paulownia fortunei*, *HSF* gene family, drought stress, gene function

## Abstract

Heat shock transcription factors (HSFs) act as crucial regulatory components in plant stress regulatory network and play essential roles in abiotic stress adaptation, particularly drought tolerance. Here, we identified 36 *PfHSF* gene family members in *Paulownia fortunei* through genome-wide analysis. Their chromosomal distribution, physicochemical properties, phylogenetic relationships, gene structures, conserved motifs, *cis*-acting elements, and post-translational modification sites were systematically characterized. Transcriptome-based expression profiling demonstrated distinct tissue-specific and drought-responsive expression patterns among *PfHSF* members, with *PfHSF6* being strongly up-regulated under drought. Subcellular localization assays confirmed nuclear and plasma membrane localization of PfHSF6. Transient overexpression of *PfHSF6* in tobacco significantly enhanced drought tolerance, as evidenced by alleviated wilting, a ~60% reduction in MDA content, ~2-fold increases in SOD and POD activities, and a ~67% reduction in electrolyte leakage relative to the control (*p* < 0.01); consistently, stress-responsive genes such as *NbRD29A* and *NbLEA5* were up-regulated by ~6- and ~3-fold, respectively. This study provides a comprehensive characterization of the *PfHSF* family and preliminary functional evidence for *PfHSF6*, offering candidate genes for further functional validation and potential drought-tolerance breeding in paulownia.

## 1. Introduction

Drought stress is one of the major abiotic stress factors restricting plant growth and development, which disrupts cellular osmotic balance, impairs photosynthesis and respiration, retards plant growth, induces leaf wilting, and even leads to plant mortality under severe conditions [1,2,3]. In response, plants have evolved sophisticated regulatory mechanisms to cope with stress during long-term evolution. In this complex regulatory network, transcription factors play a pivotal role in orchestrating stress responses.

Heat shock transcription factors (HSFs) are essential regulators in plant stress responses and play a central role in stress tolerance [4,5]. Typical HSF proteins contain five evolutionarily conserved domains: a DNA-binding domain that recognizes heat shock elements in target promoters, an oligomerization domain (OD) mediating homo- or hetero-oligomerization, a nuclear localization signal and a nuclear export signal that coordinate nucleocytoplasmic shuttling, and a C-terminal transcriptional activation domain (CTAD) that recruits RNA polymerase II and the basal transcription machinery [6]. Based on structural differences in the OD and CTAD, HSF family members are further classified into three subfamilies (A, B, and C). Subfamily A members harbor a complete CTAD and act primarily as activators, whereas subfamily B members lack a canonical CTAD and often serve as repressors or modulators; subfamily C members exhibit intermediate features and participate in specific regulatory networks [7]. This structural and functional diversity enables HSFs to integrate multiple stress signals and precisely regulate downstream gene expression.

The functional roles of *HSF* genes in drought stress responses have been increasingly elucidated through functional studies in diverse species. Overexpression of *TaHsfA2e-5D* in *Arabidopsis thaliana* enhanced tolerance to both heat and drought stresses by up-regulating the transcriptional expression of heat shock protein genes as well as stress-responsive genes such as *APX2* and *GolS1* [8]. Similarly, overexpression of *OsHsfB4b* exhibited a strong response to stress treatments and significantly improved drought tolerance in rice [9]. Furthermore, Zhu et al. [10] demonstrated that ectopic overexpression of *OsHSFA3* promoted drought tolerance in *Arabidopsis* by elevating endogenous abscisic acid levels and modulating polyamine levels to maintain reactive oxygen species (ROS) homeostasis. Conversely, overexpression of *ZmHsf08* led to increased ROS and malondialdehyde (MDA) in transgenic plants [11]. Collectively, these findings highlight the central and multifaceted roles of HSFs in plant stress adaptation. However, despite such progress, research on the *HSF* gene family in paulownia has not yet been reported, representing a significant gap in our understanding of stress tolerance mechanisms in this woody species.

*Paulownia fortunei* is an important fast-growing timber and ecological tree species native to China. Its wood features straight grain, uniform texture, light and soft material, and good workability, which make it suitable for manufacturing furniture and craft carvings [12]. Meanwhile, the flowers, leaves, fruits, bark and roots of paulownia contain bioactive ingredients that exhibit prominent medicinal potential in anti-tumor therapy, antibacterial and anti-inflammatory treatment, as well as the prevention and treatment of cardiovascular diseases [13]. Thus, *P. fortune* has significant economic and medicinal value. Nevertheless, its growth and wood quality are frequently impaired by drought stress. To date, no study has investigated the functional link between *HSF* genes and drought tolerance in this species, and a systematic characterization of the whole HSF family remains unavailable. To fill this gap, we performed a genome-wide identification of the HSF family in *P. fortunei*. Expression profiling under drought stress revealed that *PfHSF6* was significantly up-regulated and served as a candidate gene for drought stress. Transient overexpression of *PfHSF6* in tobacco enhanced drought tolerance, as evidenced by increased antioxidant enzyme activities and reduced oxidative damage. These results preliminarily establish that *PfHSF6* plays a potential role in the drought stress response in a heterologous system.

## 2. Results

### 2.1. Identification and Phylogenetic Analysis of PfHSF Genes

**Figure 1:**
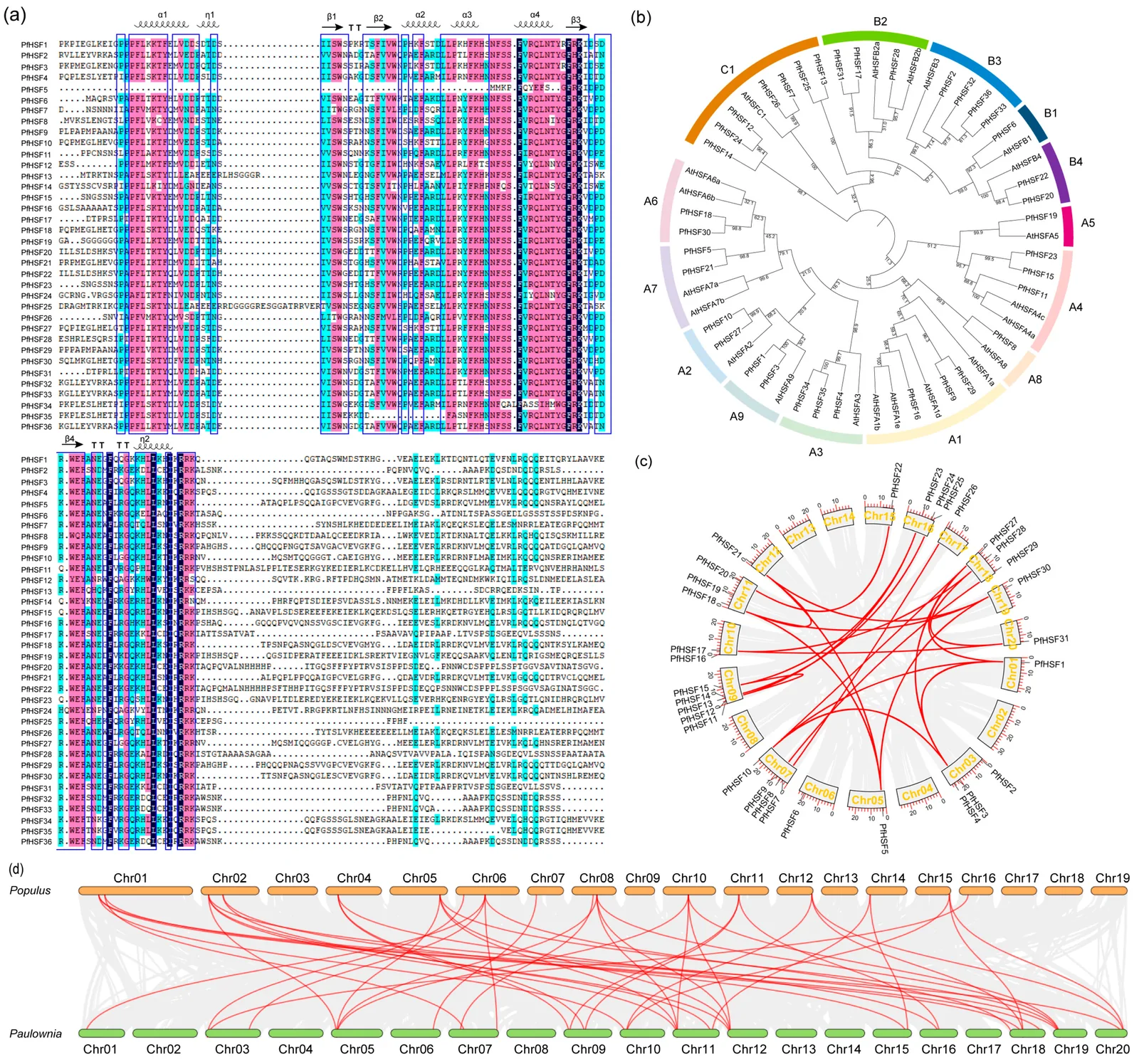
Characterization of the *PfHSF* genes. (**a**) Multiple sequence alignment of PfHSF family proteins with a typical DBD domain and conserved sequence structures. (**b**) Phylogenetic analysis of PfHSF proteins in paulownia and *Arabidopsis*. The tree revealed three HSF subfamilies, and bootstrap support values from 1000 replicates are given at branch nodes. (**c**) Intraspecific collinearity of the *PfHSF* genes. (**d**) Synteny analysis revealed the evolutionary relationships between *HSF* genes in *P. fortunei* and *P. trichocarpa*. Green and orange blocks represent the chromosomes of paulownia and poplar, respectively. The orange lines represent orthologous *HSF* pairs between species.

A total of 36 *HSF* family genes were identified, which were designated as *PfHSF1* to *PfHSF36* based on their chromosomal localization (Table S2). Chromosomal distribution analysis showed that *PfHSF* genes were unevenly anchored on paulownia chromosomes, with no members mapped on chromosomes 2, 4, 8, 13, and 14 (Figure S1). Conserved Domain Database (CDD) analyses revealed that all 36 PfHSF proteins possessed the conserved HSF-type DNA-binding domain (PF02179), a canonical structural motif that enabled specific recognition of heat shock elements and mediates transcriptional regulation of plant stress responses. Multiple sequence alignment revealed highly conserved amino acid residues within this domain, as well as retained secondary structural elements including α-helices (α1–α4), β-strands (β1–β4), and short η-helices (η1–η2) among *PfHSF* members (Figure 1a). These invariant residues were critical for domain folding and DNA-binding activity. Physicochemical property analysis revealed that the PfHSF proteins ranged from 139 to 518 amino acids in length, with molecular weights varying from 16.12 kDa to 57.45 kDa. All PfHSF proteins were predicted to be basic (pI > 7.0) and hydrophilic, with negative grand average of hydropathicity values. Subcellular localization prediction showed that all PfHSF proteins were primarily localized to the nucleus. A subset of PfHSF members additionally exhibits dual localization patterns, with signals also detected in the cytosol, plasma membrane, and other subcellular compartments, suggesting potential functional divergence within the PfHSF family.

Phylogenetic analysis classified the *PfHSF* gene family into three distinct subfamilies (Figure 1b). Subfamily A was further divided into nine subtypes (A1–A9), including 20 family members. Subfamily B was divided into four subtypes (B1–B4), including 10 family members. Subfamily C included six family members. Furthermore, intraspecific collinearity analysis identified 31 collinear gene pairs among the 36 *PfHSF* members (Figure 1c), while 46 collinear gene pairs were detected between *PfHSF* and *PtHSF* genes (Figure 1d). These results indicated that the *HSF* gene family was relatively conserved between *P. fortunei* and *P. trichocarpa*.

### 2.2. Conserved Motifs and Genomic Feature Analysis of PfHSFs

**Figure 2:**
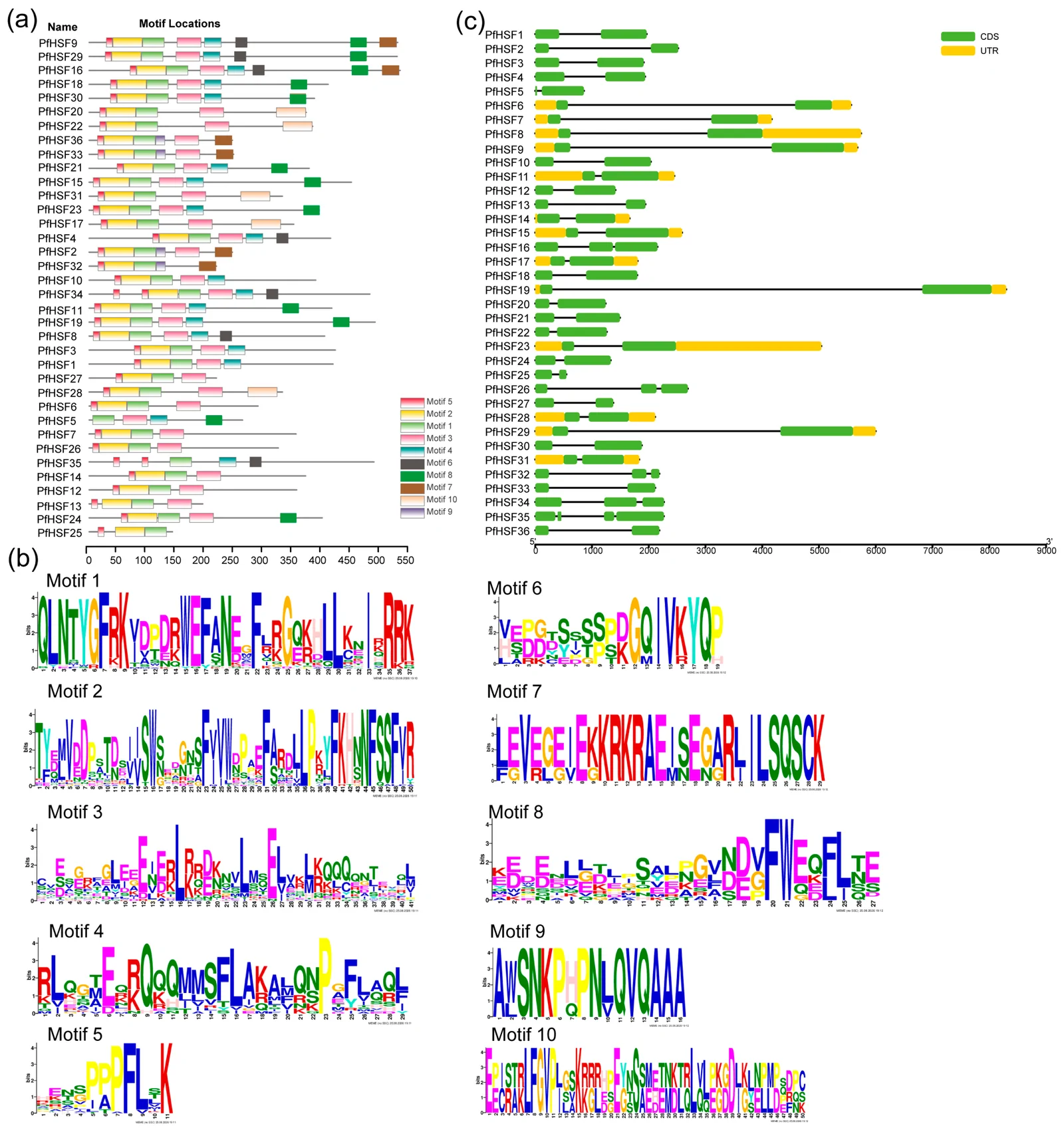
Conserved motifs and gene structure analysis of PfHSF proteins. (**a**,**b**) Identification of conserved motif architecture in PfHSF proteins. Ten conserved protein motifs (Motifs 1–10) were identified using MEME analysis (E-value < 1 × 10^−5^). Solid lines indicate protein lengths, and colored blocks represent individual motifs (**a**). Sequence logos show colored single-letter amino acids with height proportional to frequency. Numbers below and bit scores on the left denote motif length and conservation level, respectively (**b**). (**c**) Exon-intron architecture of *PfHSFs*. Exon-intron architecture was visualized with TBtools v 2.012, green blocks represent the untranslated regions, yellow blocks represent coding DNA sequences, and black lines represent intron regions.

A total of ten conserved motifs, primarily enriched in hydrophobic (V, F, L, W, G, P) and charged (E, D, R, K) residues, were identified across the 36 PfHSF proteins (Figure 2a,b), suggesting that these motifs may mediate protein–protein interactions or membrane association. Further characterization revealed that the number of motifs harbored by individual PfHSF proteins varied substantially from 2 to 8. Among them, motif 1 was ubiquitously distributed across all PfHSF members and constituted the core conserved motif of this family. In terms of evolutionary clades and motif arrangement, PfHSF members within the same phylogenetic clade shared similar motif compositions and orders. By comparison, prominent differences in motif composition were observed among members of different clades, indicating functional divergence. Gene structure analysis revealed obvious variations in exon number and gene length across different *PfHSF* members (Figure 2c). Most *PfHSF* genes contained 1–3 exons, and only a small subset possessed more than three exons. Overall, *PfHSF* members within the same clade exhibited divergent gene structures. Collectively, these results demonstrated that the *PfHSF* gene family of *P. fortunei* maintained high conservation during evolution, suggesting that the core functional domains of this family remained relatively stable throughout evolutionary processes.

### 2.3. Post-Translational Modification Sites Analysis of PfHSFs

Post-translational modification (PTM) plays a crucial role in regulating protein function. To elucidate the post-translational regulatory characteristics of the HSF family members, we systematically predicted phosphorylation and SUMOylation modification sites on PfHSF proteins (Tables S3 and S4). Phosphorylation site analysis showed that the predicted phosphorylation sites in PfHSF proteins were located on Ser, Thr, and Tyr residues. In Class-A HSF members (classified based on phylogenetic analysis and the conserved HR-A/B domain), several Ser/Thr clusters predicted to contain MAPK or CDPK recognition motifs were located upstream of the nuclear localization signal, raising the possibility that these residues could serve as kinase substrates and potentially influence nuclear localization; however, this possibility remained to be experimentally tested. SUMOylation prediction identified the canonical ΨK*x*E/D motif in PfHSF sequences, and key lysine residues of this motif were highly conserved in the transcriptional activation region. Collectively, PfHSF family members contained abundant phosphorylation and conserved SUMOylation sites, and the co-occurrence of these sites implied that phosphorylation and SUMOylation may work together in the stress-related regulation of PfHSFs. This PTM profile provided a valuable basis for future experimental validation, especially for the key drought-responsive members identified in this study.

### 2.4. Cis-Regulatory Elements in the Promoters of the PfHSF Genes

*Cis*-acting element analysis was performed on the promoter regions of all *PfHSF* genes (Table S5, Figure S2). The results revealed a widespread enrichment of stress-responsive regulatory motifs across the family, most notably ABA-responsive elements (ABREs) and MYB-binding sites (MBSs), alongside various other stress- and hormone-associated elements (e.g., TCA-element, CGTCA-motif). This broad distribution suggested that these *PfHSF* genes generally possess the regulatory capacity for transcriptional reprogramming under abiotic stress conditions. Among the *PfHSF* genes, four *PfHSF* members (*PfHSF6*, *PfHSF19*, *PfHSF34*, and *PfHSF35*) exhibited a particularly high abundance of hormone-responsive elements associated with ABA, SA, and MeJA signaling in their promoter regions. These three hormonal pathways are known to regulate key stress-adaptive processes, including stomatal regulation, osmotic adjustment, and antioxidant defense. The enrichment of their cognate cis-elements in the promoters of these four *HSF* genes suggested that they might act as downstream integrators of multiple stress-associated hormonal signals. Given the pivotal roles of ABA, SA, and MeJA in coordinating stomatal behavior, cellular osmotic balance, and oxidative stress mitigation under water-deficit conditions, the promoter profiles of *PfHSF6*, *PfHSF19*, *PfHSF34*, and *PfHSF35* suggested that these four transcription factors were responsive to stress hormonal signals and might play important roles in drought stress tolerance in *P. fortunei*.

### 2.5. Expression Pattern Analysis of the PfHSF Gene Family

**Figure 3:**
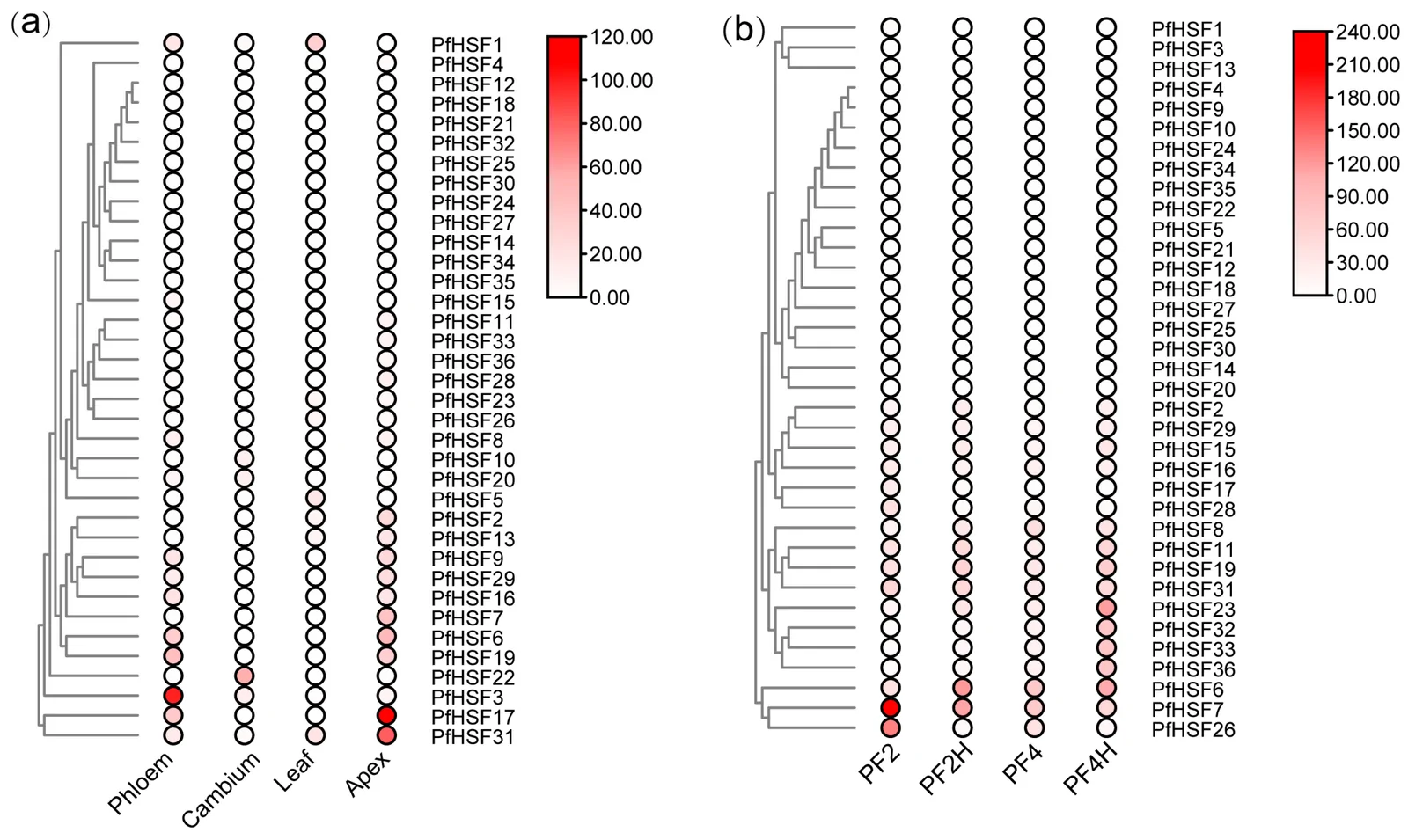
Expression pattern analysis of *PfHSF* genes. (**a**) Heatmap of normalized RNA-seq expression levels of *PfHSF* genes across four tissues in *Paulownia fortunei*. (**b**) Heatmap of normalized RNA-seq expression of *PfHSF* genes under drought stress in diploid and autotetraploid *P. fortunei*. PF2: diploid *P. fortunei*; PF2H: diploid *P. fortunei* under drought stress; PF4: autotetraploid *P. fortunei*; PF4H: autotetraploid *P. fortunei* under drought stress.

To comprehensively elucidate the biological functions of the *PfHSF* gene family in *P. fortunei*, we first analyzed their tissue-specific expression profiles across the apex, young leaf, cambium, and phloem using transcriptomic data (Figure 3a, Table S6). The analysis revealed significant divergence in expression patterns among these tissues. Specifically, *PfHSF1* exhibited preferential high expression in leaves (FPKM > 30), whereas *PfHSF3*, *PfHSF17*, and *PfHSF19* displayed peak expression levels in the phloem (FPKM > 30); notably, *PfHSF6* showed high expression in both the apex and phloem (FPKM > 30). Given that the phloem is a critical tissue for long-distance transport of osmotic adjustment substances (e.g., sugars) and stress signaling molecules (e.g., ABA), the phloem-specific high expression of these genes suggests their potential roles in mediating drought-responsive physiological processes. In addition, *PfHSF22* was preferentially expressed in cambium, while distinct expression profiles were also observed in the apex.

Subsequently, to investigate the response of the 36 *PfHSF* members to abiotic stress, we visualized their expression levels under drought treatment. The results demonstrated that most *PfHSF* genes exhibited distinct expression variations following drought stress in diploid and autotetraploid *P. fortunei* (FPKM > 30). Notably, among the highly expressed genes, five exhibited significant expression variations (fold change (FC) > 1.5 or FC < −1.5 and *p* value < 0.05) in diploid *P. fortunei* under drought stress (*PfHSF6*, *PfHSF7*, *PfHSF23*, *PfHSF26*, and *PfHSF28*) (Figure 3b, Table S7). Similarly, in autotetraploid *P. fortunei*, six highly expressed genes showed significant expression variations (FC > 1.5 or FC < −1.5 and *p* value < 0.05) (*PfHSF6*, *PfHSF11*, *PfHSF19*, *PfHSF23*, *PfHSF26*, and *PfHSF31*) (Figure 3b, Table S7). Combining these two datasets, we prioritized genes that exhibited both tissue-specific high expression (FPKM > 30) and significant drought-responsive variation (FC > 1.5 or FC < −1.5 and *p* value < 0.05). This approach identified *PfHSF6* and *PfHSF26* as key candidate genes involved in drought stress response. Among these two genes, *PfHSF26* was down-regulated under drought stress, while *PfHSF6* was up-regulated. Therefore, *PfHSF6* was considered as the key gene in response to drought stress.

**Figure 4:**
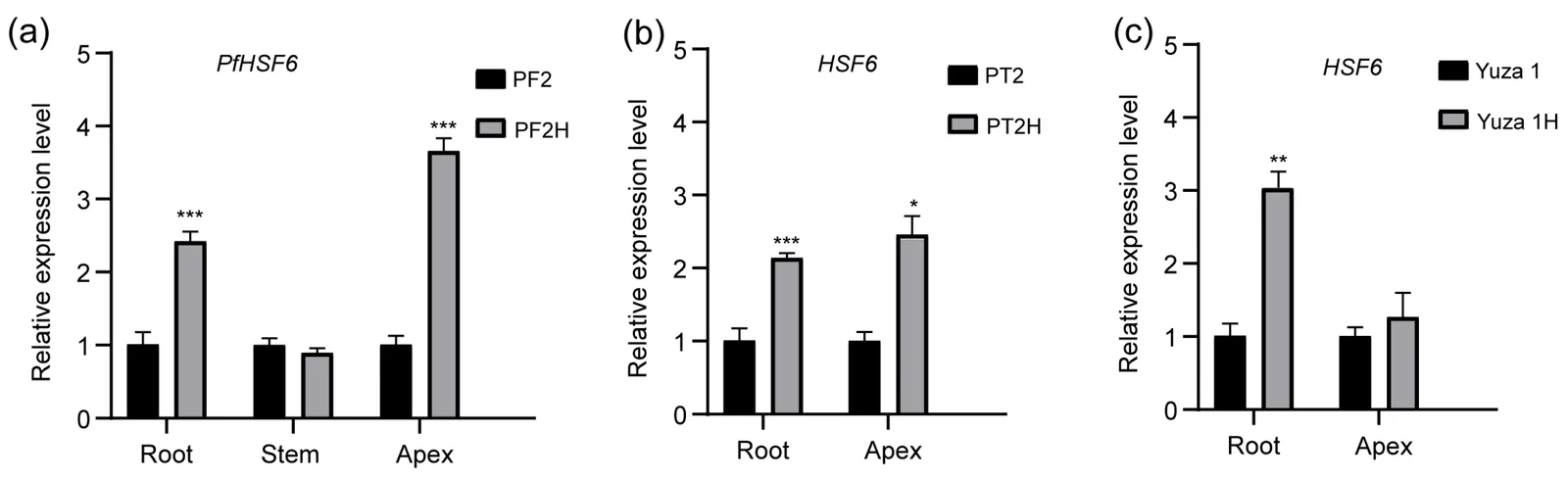
Expression analysis of *HSF6* under drought stress in different paulownia genotypes. (**a**). Quantitative RT-PCR analysis of *PfHSF6* in three tissues under drought stress. PF2: diploid *P. fortunei*; PF2H: diploid *P. fortunei* under drought stress. (**b**,**c**). Quantitative RT-PCR analysis of *PfHSF6* in two tissues under drought stress in *P. tomentosa* (**b**) and cultivar ‘*Yuza* 1’ (**c**). PT2: diploid *P. tomentosa*; PT2H: diploid *P. tomentosa* under drought stress; Yuza 1: diploid cultivar ‘*Yuza* 1’; Yuza 1H: diploid cultivar ‘*Yuza* 1’ under drought stress. Values are means ± SD from three biological replicates, and statistical differences between samples are analyzed using a two-tailed unpaired *t*-test (* *p* < 0.05, ** *p* < 0.01, *** *p* < 0.001).

To validate these findings, qRT-PCR was performed on apex, root, and stem tissues (Figure 4a). The validation results confirmed the expression patterns observed in the transcriptome data. Additionally, we verified the expression levels of *PfHSF6* under drought stress in *P. tomentosa* and cultivar ‘*Yuza* 1’ (Figure 4b,c), and the results were consistent with those in *P. fortunei*. Consequently, based on its robust expression under drought stress and verification in key tissues, *PfHSF6* was identified as the key candidate gene closely associated with drought stress in *P. fortunei*.

### 2.6. Subcellular Localization of PfHSF6 and the Effects of Its Overexpression in Tobacco

**Figure 5:**
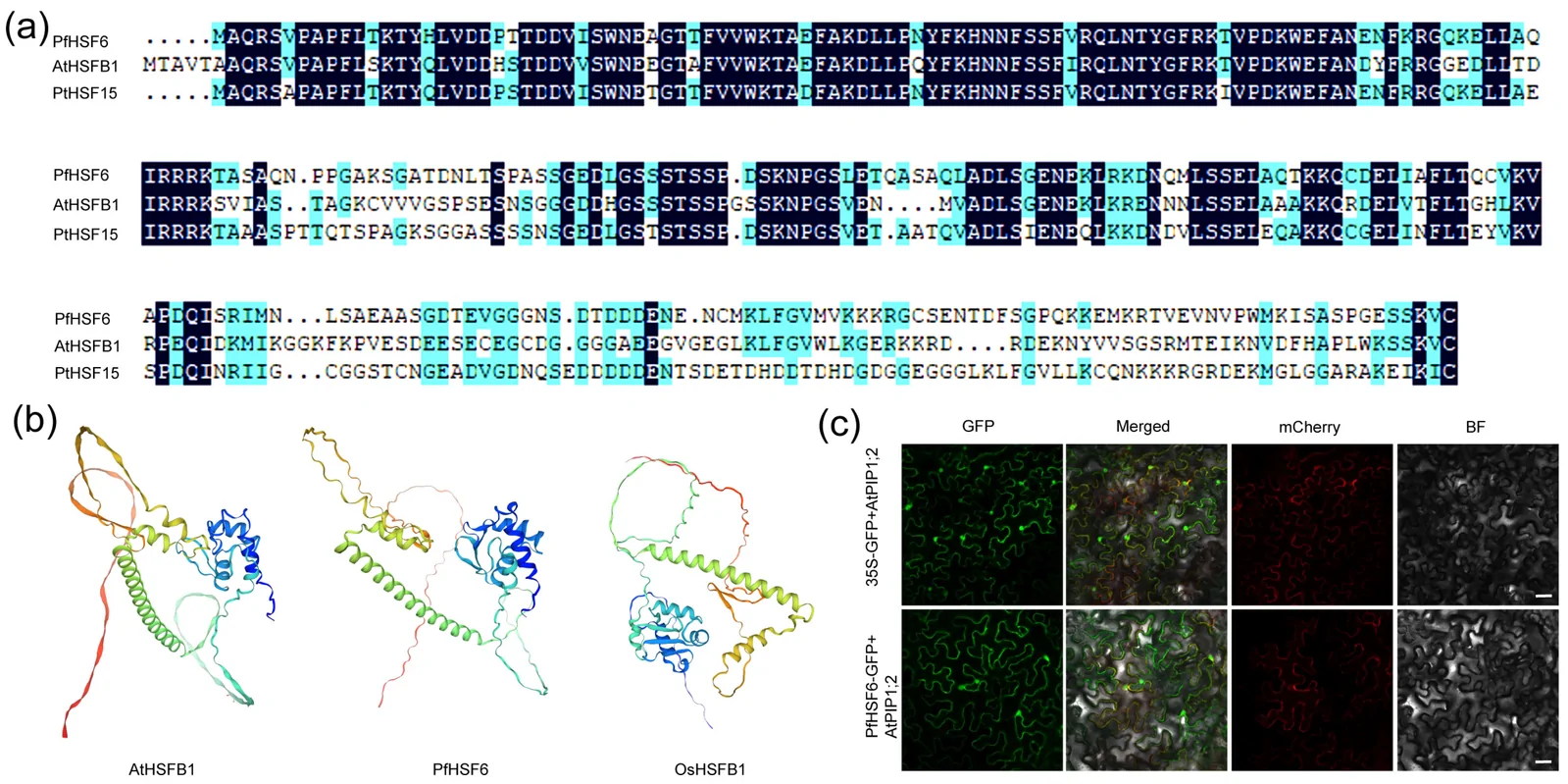
Subcellular localization analysis of PfHSF6. (**a**) Alignment of amino acid sequences of PfHSF6 from *Arabidopsis*, paulownia, and rice. DNAMAN was used for multiple sequence alignment and visualization, and amino acids shaded in black represent identical residues. (**b**) Comparative three-dimensional structural analysis of HSF proteins. Homology models of AtHSFB1, OsHSFB1 and PfHSF6 were constructed via SWISS-MODEL. (**c**) Subcellular localization of PfHSF6. The PfHSF6-GFP fusion protein transiently overexpressed in *N. benthamiana* epidermal cells is shown. The fluorescence of the fusion protein overlapped with the plasma membrane marker AtPIP1;2, indicating that PfHSF6 was localized to the plasma membrane and nucleus. Bar = 20 μm.

**Figure 6:**
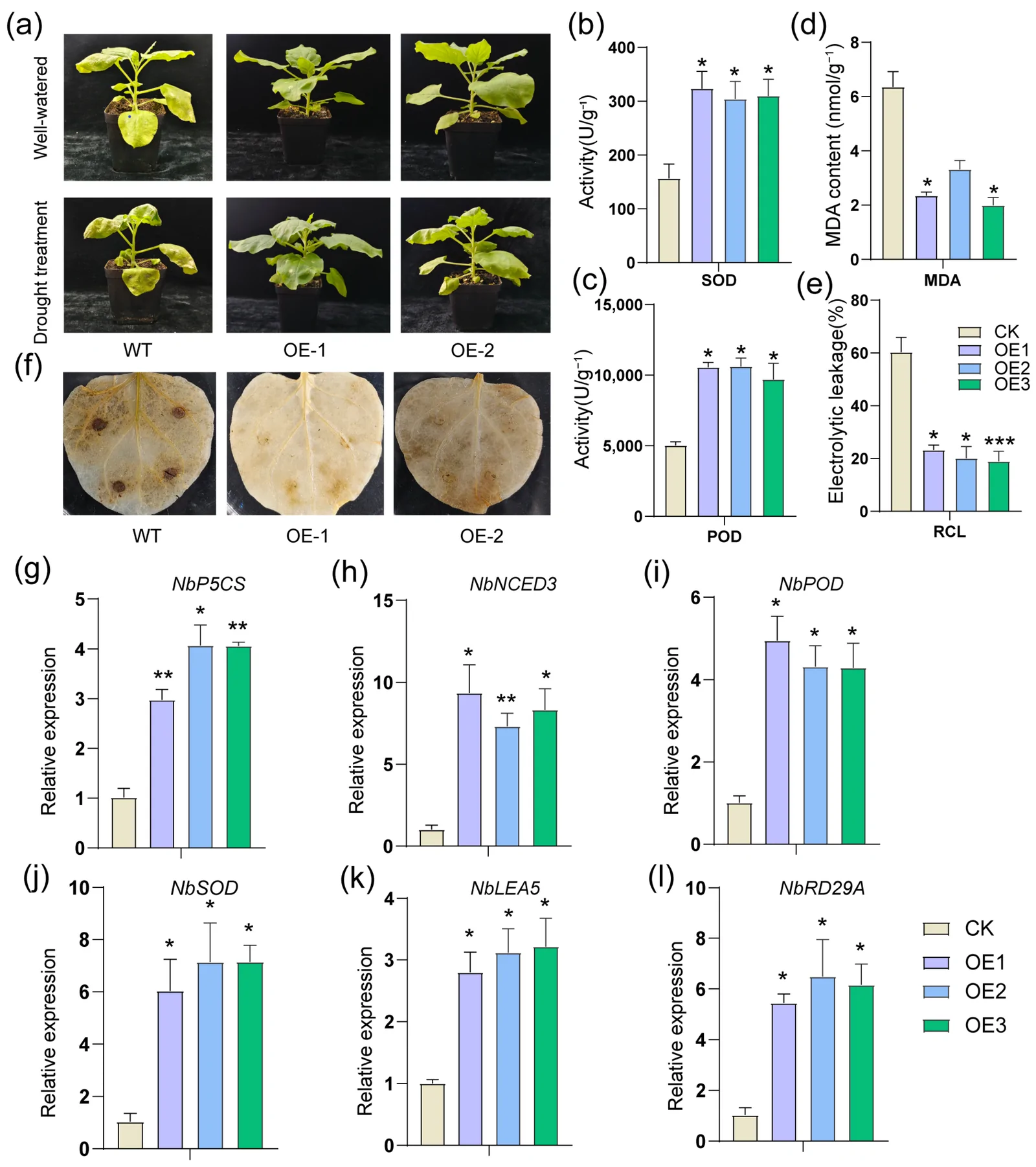
Functional analysis of *PfHSF6* via transient overexpression in tobacco (*Nicotiana benthamiana)*. (**a**) Transient expression of *PfHSF6* enhances tolerance to drought stress in tobacco. WT, wild type; OE-1 and OE-2, independent transiently overexpressing *PfHSF6* lines. Plants were photographed at 6 DAT (days after treatment). Bar = 2 cm. (**b**–**d**) Superoxide dismutase (SOD) activity (**b**), peroxidase (POD) activity (**c**), and malondialdehyde (MDA) content (**d**) under drought stress in tobacco. (**e**) The relative electrolyte leakage under drought stress. (**f**) DAB staining for ROS accumulation in *PfHSF6* transient expression leaves. (**g**–**l**) Expression levels of drought-related marker genes in transiently overexpressing lines and the WT. Data presented were means of three biological replicates with standard deviation (*n* = 3). Statistical significance was assessed by one-way ANOVA followed by Dunnett’s multiple comparisons test against the WT control. Asterisks indicate significant differences compared with the WT (* *p* < 0.05, ** *p* < 0.01, *** *p* < 0.001).

To characterize *PfHSF6* functions in response to drought stress, we cloned its full-length coding sequence (CDS) from *P. fortunei*. Sequence analysis revealed that *PfHSF6* encodes a protein of 283 amino acids with a predicted molecular weight of approximately 31 kDa (Figure S3). Homology analysis showed that *PfHSF6* shares high amino acid sequence identity with HSF homologs from *A. thaliana* (49% identity) and *P. trichocarpa* (59% identity) (Figure 5a,b). Subcellular localization assays revealed that the PfHSF6-GFP signal was predominantly detected in the nucleus and plasma membrane (Figure 5c), which is consistent with its putative function as a transcription factor. To confirm the hypothesized positive regulatory role of *PfHSF6* on drought stress, the fusion protein PfHSF6-GFP was transiently over-expressed in *N. benthamiana* leaves, then the plants were subjected to PEG-simulated drought stress at 3 days after treatment (DAT). At 6 DAT, the plants expressing the pRI101-GFP control alone showed severe wilting, whereas the *PfHSF6*-overexpressing plants remained relatively normal (Figure 6a). Furthermore, physiological measurements at 5 DAT demonstrated that transient over-expression of *PfHSF6* significantly enhanced the drought tolerance of *N. benthamiana*. Specifically, *PfHSF6*-overexpressing plants exhibited ~2-fold higher SOD and POD activities and a ~60% lower MDA content, compared to the wild type (WT) (Figure 6b–d; *p* < 0.05). Additionally, the relative electrolyte leakage was reduced by ~67% in the over-expression lines (Figure 6e; *p* < 0.05). Moreover, reactive oxygen species (ROS) accumulation, as revealed by DAB staining, was markedly reduced in the *PfHSF6*-overexpressing lines compared with the control (Figure 6f). Consistent with these physiological improvements, qRT-PCR analysis revealed that the transcript levels of several stress-responsive genes, including *NbRD29A*, *NbPOD*, *NbSOD*, *NbNCED3*, *NbP5CS*, and *NbLEA5*, were significantly up-regulated in the *PfHSF6*-overexpressing lines compared with the WT (Figure 6g–l), suggesting that these genes are co-expressed with, or potentially downstream of, *PfHSF6*. These results collectively suggest that *PfHSF6* can transcriptionally activate these stress-responsive genes in the heterologous tobacco system.

## 3. Discussion

HSFs are central regulators of plant abiotic stress responses [14,15]. Although *HSF* families have been systematically characterized and implicated in drought responses in several plant species [10,16,17], their roles in the drought response of *P. fortunei* remain unknown. Here, we performed the first genome-wide identification of the *PfHSF* gene family and systematically analyzed its phylogenetic relationships and drought-responsive expression patterns, which not only helped fill the gap in *HSF* research in paulownia but also identified key drought-responsive candidates, providing valuable genetic resources and target genes for molecular breeding of drought-tolerant paulownia.

A total of 36 *PfHSF* gene family members were identified from *P. fortunei*, which is higher than those reported in *A. thaliana* (21) [18], *Oryza sativa* (25) [19], and *Populus trichocarpa* (30) [20]. Based on our previous genomic data, the *P. fortunei* genome contains 31,985 protein-coding genes [21], giving a relative PfHSF family abundance of 0.1126% (36 members/31,985 genes). After normalizing to the total number of protein-coding genes from each reference genome, the relative abundance of *AtHSFs* was calculated as 0.0766% for *A. thaliana* [22], 0.0609% for *O. sativa* [23], and 0.0680% for *P. trichocarpa* [24]. This comparatively higher relative abundance of HSF genes in *P. fortunei* suggests that this family may have undergone lineage-specific expansion. Further collinearity analysis uncovered 31 collinear gene pairs among the 36 PfHSF members (Figure 1c) and 46 collinear gene pairs between *PfHSF* and *PtHSF* genes (Figure 1d), indicating that the HSF family is relatively conserved between *P. fortunei* and *P. trichocarpa*. Future studies including additional woody species will help further clarify the evolutionary patterns of the HSF family.

Protein phosphorylation represents a key post-translational regulatory mechanism in plant stress signaling, enabling activated kinases to modulate transcription factor activity, subcellular localization, and protein stability, thereby facilitating rapid reprogramming of stress-responsive gene expression [25,26,27]. In the present study, phosphorylation site prediction revealed that PfHSF6 harbors multiple phosphorylation sites. This prediction is noteworthy, as MAPK cascades are activated by drought and other abiotic stresses [28,29], and MAPK6-mediated phosphorylation of AtHsfA2 at Thr249 has been shown to govern its nuclear import and stability [30]; similarly, MPK3/MPK4/MPK6 directly phosphorylate HSFA6a at multiple serine residues, differentially affecting its DNA-binding activity in rice [31]. Notably, Ser120 of PfHSF6 was predicted as a potential target site for MAPKs, suggesting drought-activated MAPK cascades may target S120 to modulate the protein stability or transactivation activity of PfHSF6. Additionally, promoter *cis*-element analysis revealed that the PfHSF6 promoter harbors multiple hormone-responsive motifs, including ABRE and MeJA-responsive elements, suggesting that *PfHSF6* may be transcriptionally regulated by both ABA- and MeJA-dependent signaling pathways. The coexistence of these regulatory features points to a dual-layer control of PfHSF6 at both the post-translational and transcriptional levels during drought stress. Collectively, current evidence suggests that *PfHSF6* may play a positive regulatory role in the drought response of *P. fortunei*, and this function requires further functional validation. In addition, whether this role is mediated through an S120 phosphorylation-dependent mechanism remains to be clarified by future experiments, including in vivo phosphorylation assays, site-directed mutagenesis, and stable transgenic approaches.

The functional relevance of *PfHSF6* in drought responses was experimentally assessed through transient overexpression in tobacco. Heterologous expression of *PfHSF6* significantly alleviated drought-induced wilting, enhanced antioxidant enzyme activities, reduced oxidative damage, and maintained membrane integrity, indicating that *PfHSF6* can modulate multiple downstream drought-defense pathways. This functional role is further supported by the up-regulation of endogenous tobacco marker genes (*NbRD29A*, *NbPOD*, *NbSOD*, *NbNCED3*, *NbP5CS*, and *NbLEA5*) upon *PfHSF6* expression, confirming that it is capable of transactivating downstream genes in a heterologous system. These observations are consistent with previous reports that nucleus-localized HSFs, such as *RhHSF17* [32] and *TaHSFC3B* [33], function as positive regulators of drought tolerance. However, the heterologous transient expression system cannot fully recapitulate the native regulatory context of *P. fortunei*. Moreover, the up-regulated marker genes are tobacco orthologs, and their induction does not conclusively identify direct targets in paulownia. Future work using stable transgenic paulownia lines, combined with transcriptomic and chromatin-binding approaches, will be essential to delineate the downstream regulatory network of *PfHSF6* and to evaluate its drought-inducible expression pattern and functional relevance under native conditions.

## 4. Materials and Methods

### 4.1. Plant Material

Paulownia material used in this study was obtained from the Paulownia Biology Laboratory of Henan Agricultural University, China. Tissue-cultured plantlets of diploid *P. fortunei*, *P. tomentosa*, the cultivar ‘*Yuza 1*’, and autotetraploid *P. fortunei* were grown for 30 days in a greenhouse at 25 ± 2 °C. For drought treatment, uniform seedlings were carefully uprooted, and their root systems were individually immersed in 15% Polyethylene glycol 6000 (PEG 6000). After 24 h, the apex, root, and stem were collected. For each treatment, samples were pooled from five individual plants, with three biological replicates per treatment. All samples were immediately frozen in liquid nitrogen and stored at −80 °C until RNA extraction.

### 4.2. Identification and Bioinformatic Analyses of Putative PfHSF Genes

To identify PfHSF homologs in the *P. fortunei* genome, a comprehensive strategy combining BLASTP searches (E-value < 1 × 10^−5^, coverage ≥ 50%, identity ≥ 30%) and HMMER searches (PF00447 domain, E-value < 1 × 10^−5^) was employed using a stratified pipeline based on the refined *Arabidopsis* HSF dataset from TAIR. Candidate genes were validated via CDD for domain integrity. The online ExPASy ProtParam server (https://web.expasy.org/protparam/, accessed on 6 September 2026) and Wolf PSORT (https://wolfpsort.hgc.jp/, accessed on 6 September 2026) were used to analyze physicochemical properties and predict subcellular localization, respectively.

To construct the phylogenetic relationships of PfHSF, maximum-likelihood trees were constructed. Protein sequences from *P. fortunei* and *A. thaliana* were aligned using MAFFT (v7.407), and the resulting alignments were trimmed with trimAl (v1.4.rev22). All trees were reconstructed in IQ-TREE (v3.0.1), with the best-fit substitution model selected by ModelFinder and branch support assessed with 1000 ultrafast bootstrap replicates.

Gene structure and chromosome location were analyzed using TBtools (v 2.012) [34]. MEME was used to identify conserved motifs of PfHSFs, with parameters set as follows: number of motifs = 10, width range of motif = 6–50 amino acids, and E-value < 0.05 [35].

Collinear gene pairs of *HSFs* were identified using the MCScanX algorithm and visualized with TBtools v2.23 [11] under default parameters requiring ≥ 5 gene pairs per block and a BLAST v2.23 E-value cutoff < 1 × 10^−5^. The promoter sequences (~2000 bp) upstream of the transcription start site of all PfHSFs were retrieved from paulownia genome using TBtools, and cis-regulatory elements were predicted using PlantCARE (http://bioinformatics.psb.ugent.be/webtools/plantcare/html/, accessed on 6 September 2026). The tertiary structure was predicted using SWISS-MODEL (https://swissmodel.expasy.org/, accessed on 6 September 2026).

### 4.3. Post-Translational Modification Site Analysis of PfHSFs

The phosphorylation and SUMOylation modification sites of PfHSFs were predicted with default parameters using NetPhos–3.1 (https://services.healthtech.dtu.dk/services/NetPhos–3.1/, accessed on 6 September 2026) and JASSA v4 (http://www.jassa.fr/index.php?m=jassa, accessed on 6 September 2026), respectively.

### 4.4. Expression Analysis of PfHSFs

For expression analysis, transcriptome data of apexes, leaves, internodes, and nodes of *P. fortunei* were obtained from our previous study (NCBI accession numbers SRX11787882–SRX11787971). Transcriptome data from drought-treated diploid and autotetraploid *P. fortunei* seedlings were obtained from a previously published report (NCBI accession numbers SRX1153273–SRX1153476) [36]. The relative expression levels of each *PfHSF* gene were normalized using Fragments per kilobase of exon per million mapped reads (FPKM) values. A heatmap based on FPKM values was generated with TBtools software (version 2.119).

For gene expression validation, RT-qPCR was applied. Total RNA was extracted using an RNA Extraction Kit (Vazyme, Nanjing, China). cDNA was synthesized using the PrimeScript™ Fast RT Reagent Kit with gDNA Eraser (TaKaRa, Tokyo, Japan). *PfGAPDH* was used as the reference gene. The relative expression level was calculated using the 2^−∆∆CT^ method with three biological replicates [37]. Gene-specific primers are listed in Table S1.

### 4.5. Subcellular Localization

The coding sequence of *PfHSF6* was cloned into the pRI101-GFP expression vecto^r^ [38]. Positive *Agrobacterium tumefaciens* single colonies harbouring the *PfHSF6-GFP* construct were cultured and resuspended in infiltration buffer (10 mM MgCl_2_, 10 mM MES, pH 5.6, 150 µM acetosyringone) to an OD_600_ of 0.6. The suspension was co-infiltrated into leaves of three-week-old *N. benthamiana* together with the plasma membrane-marker construct *AtPIP1;2*. At 72 h post-infiltration, GFP fluorescence signals were visualized using a Zeiss LSM710 confocal laser-scanning microscope (Carl Zeiss AG, Oberkochen, Germany) with 488 nm excitation.

### 4.6. Transient Overexpression of PfHSF6 in Tobacco

For functional validation of *PfHSF6*, the recombinant pRI101-PfHSF6-GFP fusion expression vector and empty pRI101-GFP control vector were independently transformed into *A. tumefaciens* strain GV3101. The agroinfiltration assay was conducted in *N. benthamiana* according to a previously established transient expression protocol with minor modifications [39]. The infiltrated tobacco seedlings were incubated under normal growth conditions for 72 h to facilitate the stable accumulation of recombinant PfHSF6-GFP protein. For simulated drought stress treatment, the tobacco seedlings were fully irrigated with 15% PEG6000 solution for mimicking physiological drought stress [40]. Three days after PEG 6000 treatment, leaf phenotypic symptoms were observed. To evaluate drought-induced cell damage, toluidine blue staining was performed. Subsequently, leaf samples were collected for the determination of marker gene expression analysis, and *NbActin* was used as reference. The enzymatic activities of SOD, POD, and MDA content were determined as previously described [41]. The relative electrolyte leakage was measured as reported by Shi et al. [42]. All physiological measurements were performed with three independent biological replicates.

## 5. Conclusions

This study provides the first genome-wide systematic characterization of the *HSF* gene family in *P. fortunei*. A total of 36 *PfHSF* genes were identified and phylogenetically categorized into three conserved subfamilies. Comparative genomic analysis revealed lineage-specific expansion of the *HSF* family in *P. fortunei* relative to herbaceous species, as well as high sequence collinearity with *P. trichocarpa*, supporting the evolutionary conservation of this family across woody plants. Comprehensive bioinformatic profiling further described their structural features, post-translational modification sites and *cis*-regulatory element repertoires, laying a foundational framework for functional investigation of *HSFs* in woody species. Tissue-specific and drought-responsive transcriptome analyses identified distinct expression patterns across the *PfHSF* family. Notably, *PfHSF6* was consistently upregulated under drought stress across multiple paulownia genotypes and tissues, and its promoter harbored abundant stress- and hormone-responsive *cis*-elements, marking it as a key candidate associated with drought stress response. Preliminary functional assessment via transient heterologous overexpression in *N. benthamiana* indicated a potential positive effect of *PfHSF6* on drought stress resilience, including enhanced antioxidant enzyme activities, reduced reactive oxygen species accumulation and lipid peroxidation, and induced expression of stress-responsive marker genes. Overall, this work fills the research gap of genome-wide HSF family characterization in *P. fortunei*, and provides candidate genes and baseline data for further exploration of *HSF*-mediated drought adaptation mechanisms in woody plants. Further studies using native host functional assays are required to clarify the exact regulatory role of *PfHSF6* and assess its application potential in paulownia drought-tolerance breeding.

## Data Availability

The datasets analyzed in this study are publicly available from NCBI (accession numbers: SRX11787882-SRX11787981 and SRX: 1153473-SRX1153476).

## Supplementary Materials

The following supporting information can be downloaded at https://www.mdpi.com/article/10.3390/ijms27209338/s1.

## Abbreviations

The following abbreviations are used in this manuscript:

CD	Oligomerization domain
CDD	Conserved domain database
CDS	Coding sequence
CTAD	C-terminal transcriptional activation domain
FPKM	Fragments per kilobase of exon per million mapped reads
GFP	Green fluorescent protein
HSF	Heat shock transcription factors
MDA	Malondialdehyde
PEG	Polyethylene glycol
POD	Peroxidase
ROS	Reactive oxygen species
RT-qPCR	Real-Time quantitative PCR
SOD	Superoxide dismutase
