# Supplementary material for: Genome-Wide Characterization of the HSF Gene Family in Paulownia fortunei and Preliminary Functional Analysis of PfHSF6 in Drought Response
Source: Int J Mol Sci. 2026 Oct 21;27(20):9338. doi: 10.3390/ijms27209338 (PMC13705143; doi:10.3390/ijms27209338)
Supplement: Supplementary file 1 [file ijms-27-09338-s001.zip › Supplemantary Figures.pdf]

Supplementry Figure Legends

Supplementary Figure S1 Chromosomal distribution of PfHSF genes. Chromosomal positions are denoted by black scale bars.

Supplementary Figure S2 Cis-regulatory element distribution in *PfHSF* promoters. Promoter regions (2,000 bp upstream) of *PfHSF* genes were screened using the PlantCARE database.

Supplementary Figure S3 Sequence of PfHSF6 gene. Asterisk indicates the stop codon.

Figure S1

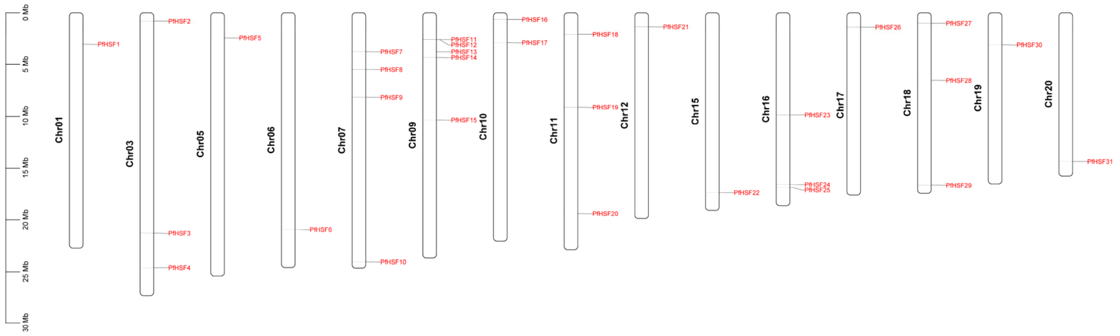
0 Mb
5 Mb
10 Mb
15 Mb
20 Mb
25 Mb
30 Mb
Chr01
Chr03
Chr05
Chr06
Chr07
Chr09
Chr10
Chr11
Chr12
Chr15
Chr16
Chr17
Chr18
Chr19
Chr20
PfHSF1
PfHSF2
PfHSF3
PfHSF4
PfHSF5
PfHSF6
PfHSF7
PfHSF8
PfHSF9
PfHSF10
PfHSF11
PfHSF12
PfHSF13
PfHSF14
PfHSF15
PfHSF16
PfHSF17
PfHSF18
PfHSF19
PfHSF20
PfHSF21
PfHSF22
PfHSF23
PfHSF24
PfHSF25
PfHSF26
PfHSF27
PfHSF28
PfHSF29
PfHSF30
PfHSF31

Figure S2

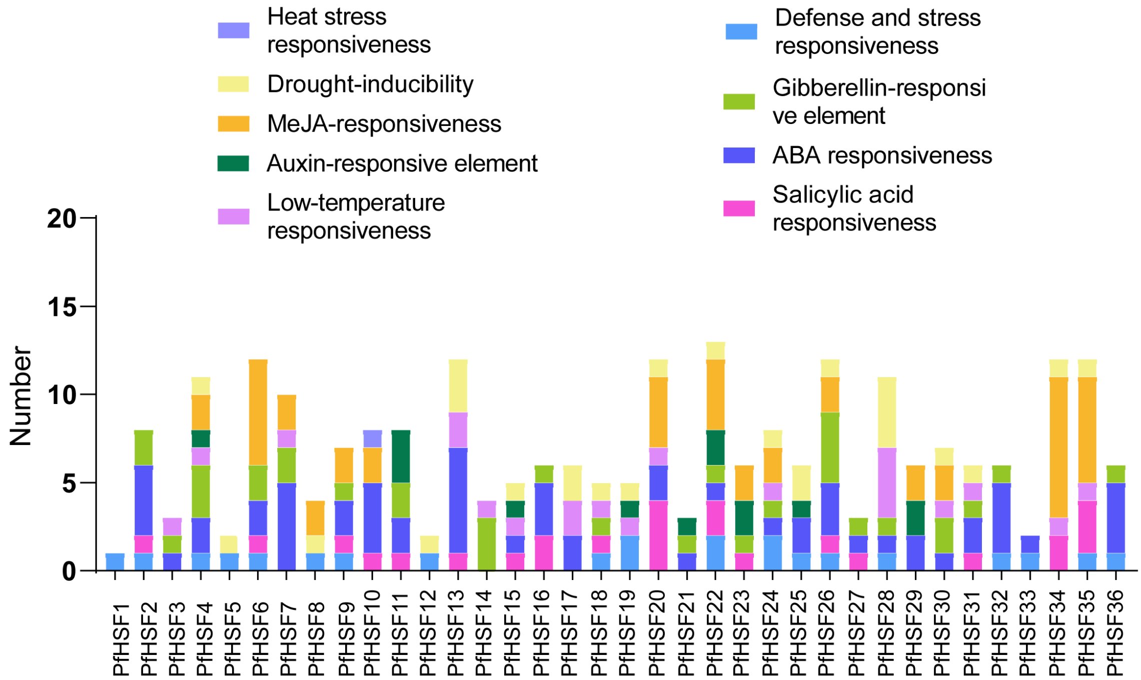
Heat stress responsiveness
Drought-inducibility
MeJA-responsiveness
Auxin-responsive element
Low-temperature responsiveness
Defense and stress responsiveness
Gibberellin-responsive element
ABA responsiveness
Salicylic acid responsiveness
Number
0
5
10
15
20
PfHSF1
PfHSF2
PfHSF3
PfHSF4
PfHSF5
PfHSF6
PfHSF7
PfHSF8
PfHSF9
PfHSF10
PfHSF11
PfHSF12
PfHSF13
PfHSF14
PfHSF15
PfHSF16
PfHSF17
PfHSF18
PfHSF19
PfHSF20
PfHSF21
PfHSF22
PfHSF23
PfHSF24
PfHSF25
PfHSF26
PfHSF27
PfHSF28
PfHSF29
PfHSF30
PfHSF31
PfHSF32
PfHSF33
PfHSF34
PfHSF35
PfHSF36

Figure S3

```
5' ATGGCGCAAAGGTCTGTTCCGGCGCCGTTTCTGACGAAGACGTATCATTTGGTGGATGATCCGACCACCGACGACGTGATCTCGTGGAACGAGGC
   M  A  Q  R  S  V  P  A  P  F  L  T  K  T  Y  H  L  V  D  D  P  T  T  D  D  V  I  S  W  N  E  A

TGGTACGACGTTTGTGGTCTGGAAAACTGCGGAATTCGCTAAGGATTTACTGCCGAATTACTTCAAGCACAACAATTTCTCCAGCTTCGTTCGCC
 G  T  T  F  V  V  W  K  T  A  E  F  A  K  D  L  L  P  N  Y  F  K  H  N  N  F  S  S  F  V  R

AGCTCAATACCTATGGTTTTCGAAAAACTGTTCCTGATAAATGGGAATTCGCCAACGAAAACTTCAAGCGAGGACAGAAGGAGCTCCTAGCGCAA
Q  L  N  T  Y  G  F  R  K  T  V  P  D  K  W  E  F  A  N  E  N  F  K  R  G  Q  K  E  L  L  A  Q

ATTCGCCGCCGGAAAACGGCATCCGCTCAGAATCCACCCGGCGCGAAGTCTGGAGCCACAGATAATCTAACCTCACCAGCAAGCTCCGGCGAAGA
  I  R  R  R  K  T  A  S  A  Q  N  P  P  G  A  K  S  G  A  T  D  N  L  T  S  P  A  S  S  G  E  D

CTTGGGTTCGAGCTCCACCTCCTCTCCTGATTCCAAAAACCCTGGCTCATTGGAGACACAGGCATCGGCGCAGCTCGCCGACTTATCCGGTGAGA
 L  G  S  S  S  T  S  S  P  D  S  K  N  P  G  S  L  E  T  Q  A  S  A  Q  L  A  D  L  S  G  E

ACGAAAAGCTGAGAAAGGATAATCAAATGCTCAGCTCCGAGCTTGCACAAACCAAGAAACAATGCGATGAGCTGATAGCTTTTTTGACTCAGTGC
N  E  K  L  R  K  D  N  Q  M  L  S  S  E  L  A  Q  T  K  K  Q  C  D  E  L  I  A  F  L  T  Q  C

GTGAAGGTGGCGCCTGACCAAATCAGCCGTATTATGAATCTCAGCGCCGAGGCTGCCAGCGGCGACACGGAGGTGGGTGGTGGAAATTCTGACAC
 V  K  V  A  P  D  Q  I  S  R  I  M  N  L  S  A  E  A  A  S  G  D  T  E  V  G  G  G  N  S  D  T

TGATGATGACGAAAATGAAAATTGTATGAAATTGTTTGGGGTGATGGTGAAAAAGAAGAGGGGTTGTAGCGAGAATACTGATTTCTCTGGGCCAC
 D  D  D  E  N  E  N  C  M  K  L  F  G  V  M  V  K  K  K  R  G  C  S  E  N  T  D  F  S  G  P

AAAAGAAAGAAATGAAAAGGACTGTGGAGGTTAATGTGCCTTGGATGAAGATTTCTGCGTCACCGGGGGAGAGCAGCAAGGTCTGTAACTGA 3'
Q  K  K  E  M  K  R  T  V  E  V  N  V  P  W  M  K  I  S  A  S  P  G  E  S  S  K  V  C  N  *
```
